# Cancer Microenvironment: What Can We Learn from the Stem Cell Niche

**DOI:** 10.3390/ijms161024094

**Published:** 2015-10-12

**Authors:** Lukas Lacina, Jan Plzak, Ondrej Kodet, Pavol Szabo, Martin Chovanec, Barbora Dvorankova, Karel Smetana

**Affiliations:** 1 Institute of Anatomy, 1st Faculty of Medicine, Charles University, U Nemocnice 3, 12800 Prague 2, Czech Republic; E-Mails: lukas.lacina@lf1.cuni.cz (L.L.); ondrej.kodet@lf1.cuni.cz (O.K.); pavol.szabo@lf1.cuni.cz (P.S.); bdvorankova@seznam.cz (B.D.); 2 Department of Dermatology and Venereology, 1st Faculty of Medicine and General University Hospital, Charles University, U Nemocnice 2, 12808 Prague 2, Czech Republic; 3 Department of Otorhinolaryngology and Head and Neck Surgery, 1st Faculty of Medicine and University Hospital Motol, Charles University, V Úvalu 84, 15006 Prague 5, Czech Republic; E-Mails: jan.plzak@lf1.cuni.cz (J.P.); martin.chovanec@lf1.cuni.cz (M.C.)

**Keywords:** stem cell, niche, wound healing, cancer microenvironment, cancer-associated fibroblast

## Abstract

Epidermal stem cells (ESCs) are crucial for maintenance and self- renewal of skin epithelium and also for regular hair cycling. Their role in wound healing is also indispensable. ESCs reside in a defined outer root sheath portion of hair follicle—also known as the bulge region. ECS are also found between basal cells of the interfollicular epidermis or mucous membranes. The non-epithelial elements such as mesenchymal stem cell-like elements of dermis or surrounding adipose tissue can also contribute to this niche formation. Cancer stem cells (CSCs) participate in formation of common epithelial malignant diseases such as basal cell or squamous cell carcinoma. In this review article, we focus on the role of cancer microenvironment with emphasis on the effect of cancer-associated fibroblasts (CAFs). This model reflects various biological aspects of interaction between cancer cell and CAFs with multiple parallels to interaction of normal epidermal stem cells and their niche. The complexity of intercellular interactions within tumor stroma is depicted on example of malignant melanoma, where keratinocytes also contribute the microenvironmental landscape during early phase of tumor progression. Interactions seen in normal bulge region can therefore be an important source of information for proper understanding to melanoma. The therapeutic consequences of targeting of microenvironment in anticancer therapy and for improved wound healing are included to article.

## 1. Stem Cells: Clinical Expectation and Reality

Data presented in this article are summarizing various *in vitro* as well as animal experiments and also include published results from human clinics and pathology. It must be noted here, that the direct bench-to-bed translation can be negatively influenced by interspecies differences between humans and animal models and therefore careful and stringent interpretation is necessary.

It is broadly accepted paradigm that tissues contain certain lowly differentiated cells with importance for self-renewal and participating in the regeneration/reparation after tissue damage. These cells are usually referred as the adult tissue stem cells. According to their differentiation plasticity, we distinguish multipotent adult stem cells that can differentiate to at least two different cell lines and monopotent/progenitor stem cells that give rise to one cell line only. The stem cell research was associated with a high expectation for regenerative medicine, where multiple therapeutic procedures based on cell/tissue engineering were proposed. Unfortunately, only the hematopoietic stem cell grafting is recently in routine clinical use for the treatment of hematopoietic malignancies, certain types of immune deficiencies and after radiation accidents [1]. In principal, harvesting of bone marrow and its grafting is usually not more complicated than the routine blood transfusion. However, the treated patient requires a specific therapeutic regimen before the transplantation and also after the procedure, while the graft does not provide full functional activity yet. Of note, event highly potent myeloablative conditioning regimen leaves the non-hematopoietic components of bone marrow behind, preserving thus the microenvironment relatively unharmed. The routine application of other stem cells in clinics is not methodologically so easy. As the proportion of stem cells in human tissues (other than bone marrow) is usually low [2], the stem cells must usually be manipulated *ex vivo* first. These procedures are strictly regulated by responsible legal authorities and require special and expensive laboratory facilities [3,4].

Because the primary SC yield from the source tissue is low, their preparation on clinically relevant scale inevitably requires *in vitro* expansion. This challenging task represents a serious biological problem, because maintenance of their stemness is dependent on the specific microenvironment called niche. Multiple aspects of molecular structure of the niche were extensively studied by various authors. Unfortunately, our full understanding to complexity of niche biology has not been achieved yet [5]. From this point of view, study of microenvironment and its deregulation in pathological conditions can bring us important information, which can be translated to our clearer understanding to this aspect of normal tissue biology.

## 2. ESCs and Their Niche

Human epidermis was the first tissue prepared *in vitro*, even earlier before the concept of SC emerged in non-hematological tissue biology. Even this pioneer research clearly demonstrated the importance of microenvironment, because feeder cells such as lethally irradiated mouse 3T3 fibroblasts were necessary for successful preparation of cultured epithelial autograft sheet [6,7]. These experiments initiated extensive efforts in the treatment of traumatic or trophic skin defects, even in human patients, by autologous transplantation. Numerous modifications of the original method were developed over the following years in attempt to speed up the procedure, increase cell yield, remove antigenic substances (e.g., bovine serum) or xenogenic material (e.g., murine feeder) [8,9,10,11]. Unfortunately, the overall success of this type of therapy was not easily predictable because of various issues such as bacterial contamination or drying of the graft during the surgery and subsequent care. Interindividual variability in proliferative potential was also observed. Moreover, due to the necessity of specialized clean high-tech cultivation facility, costly equipment and labor-intensive process, unfortunately, declined the initial optimism with large scale employment of this technology in the clinics [12].

Adult tissue stem cells are characterized by very slow cell cycling and so they retain labeled precursors of DNA synthesis [13]. Their mitotic division is usually asymmetric [14] resulting in one cell with stem cell properties and the second one (transit amplifying cell) with rapid but limited proliferation capacity leading to go to the differentiation cascade. Epidermal stem cells reside in special sites important for maintenance of their correct function [15]. The cells with stem cell properties are present in the basal layer of interfollicular epidermis or related stratified mucous membranes [16], also in the bulge region of outer root sheath of hair follicle and eventually also in the sweat gland ducts [17]. The interfollicular ESCs have more character of monopotent/progenitor stem cell [18]. ESCs located in the bulge also seem to be able to differentiate to cells forming the sebaceous gland and participate in the control of hair cycle [19]. The bulge region of hair follicle seems to be a highly interesting site, because it also represents site of residence of multipotent stem cell originated from the neural crest in both human and mice, respectively [20,21]. The possible interaction between both bulge region resident stem cell types was proposed [22] and expression of transcription factor NFIB in ESCs seems to be responsible for orchestration of both stem cell types [23]. These data indicate that ESC niche must be very precisely defined because outside above-described locations ESCs lose their stem potential and terminally differentiate. The whole pilosebaceous unit from the infundibulum to the hair bulb and sebaceous gland seems to be finely compartmentalized in mouse [24,25]. This precise architecture of hair follicle with respect to the function of ESCs seems to be dependent on expression of transcription factor LHX2 and kinase Sgk3 [26,27] together with repression of Notch signaling in surrounding epithelial cells [28]. The autoimmune damage of the stem cell niche including the increased expression of chemokines receptor CXCR3 can be relevant in pathology and it may be responsible for formation of cicatricial alopecia [29], a disease where self-renewal capacity of the follicular unit is completely exhausted. Next to the epithelial cells, the dermal cells seem to participate in the formation of ESC niche too. Fibroblasts produce tenascin-W and -C, respectively, forming thus a dermal sheath surrounding epidermal appendages. This can contribute to ESC niche formation as well [30,31,32,33,34]. Similar effect was also attributed to adipocytes and their precursors with properties of mesenchymal stem cells [35,36]. These specific microenvironmental cues are responsible for “relative dormancy” status of ESCs under the physiological condition and their activation in case of need [37]. The intensive crosstalk between ESCs and other cells in their vicinity is not surprising from the developmental point of view. Classical experiments in bird embryos clearly demonstrated that formation of epidermal appendages such as scales or feathers is controlled by surrounding fibroblasts [38]. Similarly, dermal fibroblasts are able to initiate program of pilosebaceous differentiation in corneal epithelium [39]. In opposite way, the corneal limbal microenvironmental cues lead hair follicle ESCs to differentiate to usual flat corneal epithelial cells [40]. Next to biological factors (the specific cells, extracellular matrix, growth factors, cytokines), purely physical properties of the microenvironment (e.g., surface nanostructure, shear forces, rigidity, elasticity) also contribute to niche formation [41,42].

## 3. ESCs and Wound Healing

**Figure 1:**
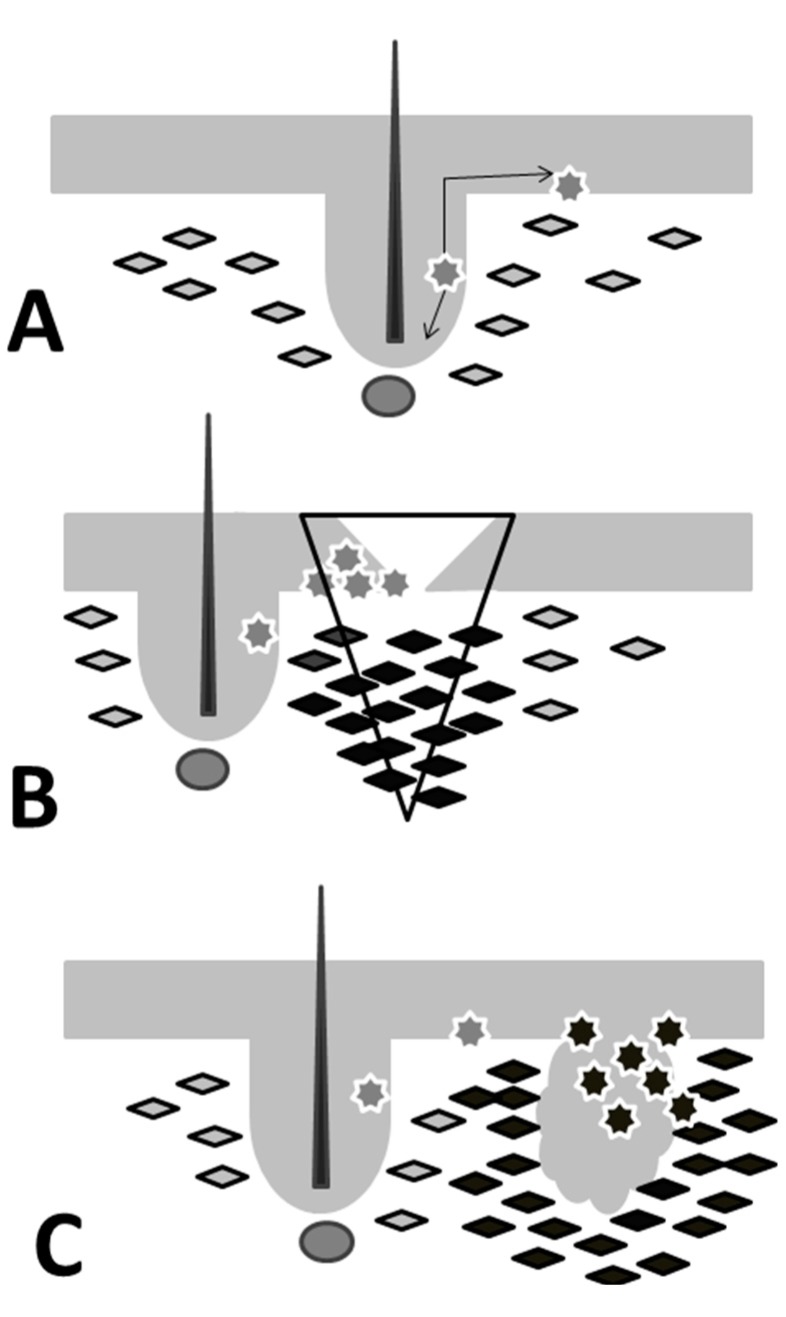
Comparison of wound healing and cancer. Multipotent epidermal stem cells are present in bulge region of hair follicle and interfollicular progenitor cells (grey asterisks) are present in basal layer of epithelium. Dermal fibroblasts (quadrangles) are separated from the epithelium by basement membrane form the functional unit with epithelial layer. (**A**) Activated stem cells, precursor cells and transit amplifying cells together with activated fibroblasts of granulation tissue (black quadrangles) participate in the closure of the wounded tissue, migration of progeny of stem cells out from the bulge region of hair follicle (arrow); (**B**) Cancer-associated fibroblasts (black quadrangles), morphologically and functionally very similar to cells from granulation tissue support the growth of cancer cells also via the stimulation of cancer stem cells (black asterisks), wound site(inverted triangle); (**C**) Activated fibroblasts and CAFs produce extracellular matrix and also the proteinases for their degradation as well as numerous bioactive cytokines/chemokines/growth factors.

Healing of wounded skin is a complex biological process including blood coagulation, infiltration of wound bed by inflammatory cells, formation of granulation tissue and wound closure [43]. Reepithelization starts from wound edges and also from the hair follicles, if partially preserved. ESCs clearly participate in the reepithelization [44,45]. They can be activated from their relative dormancy and healing will be facilitated for example by SDF-1 [46]. As described above in paragraph about the stem cell microenvironment, epithelial–mesenchymal interaction is very important in formation of ESC niche. Formation of suitable ESC niche is crucial in determination of prospective epidermal appendages. Fibroblasts, as the main cell type of granulation tissue, are indispensable for correct wound healing (Figure 1). As they produce molecules of extracellular matrix and numerous growth factors/cytokines/chemokines, they also interact with epithelial cells including stem cells and transit amplifying cells [47,48]. Introduction of mesenchymal stem cells to the wound bed can facilitate process of the skin defect healing [49]. Deregulation of the microenvironment during the wound healing results in delayed healing in venous ulcers or in diabetic foot syndrome [50,51]. Part of granulation tissue fibroblasts is transformed to the myofibroblasts due to activity of TGF-β1/3 and/or galectin-1 [52,53]. Myofibroblasts are very active in formation of extracellular matrix and contribute to the reduction of wound size by their contractibility. Stimulated fibroblasts exhibiting smooth muscle actin produce complex 3-D scaffolds rich in fibronectin and galectin-1. Keratinocytes cultured on these matrices are very small and highly express keratin 19 as a marker of low differentiation status. Stimulating effect of galectin-1 on wound healing was also confirmed in animal experiment [52]. However, abundant presence of myofibroblasts in the wound bed or in tissue stroma is proposed as a cause of hypertrophic scaring or organ fibrosis [54,55]. Remodelation of the wound bed is the last stage of the wound healing. This process can be very long and includes resorption of provisional molecules of extracellular matrix by activity of various proteases and their final replacement by collagen type I and III [43]. Multiple clinical evidences suggest that chronic wounds are accompanied by increased risk of cancer. This can be explained by prolonged exposure to inflammatory mediators such as IL-6, IL-8, CXCL-10, platelet-derived growth factor (PDGF), fibroblast growth factor-2 (FGF-2) and long lasting activation of cyclooxygenase-2 (COX-2) and matrixmetalloproteinases. Elevated activity of these factors during the unsuccessful wound healing can also participate in the cancer initiation by formation of cancer promoting microenvironment [56,57,58].

## 4. ESCs and Cancer

Existence of malignant stem cells was initially observed in hematologic malignancies and later also in solid tumors [59]. Similarly to normal adult tissue stem cells, proliferation of CSCs is very slow and they are able to readily export xenobiotics from their cytoplasm. From this point of view, CSCs are very important target in clinical oncology, because these properties minimize chance of any therapeutic hit on them. This explanation can be behind so clinically important phenomena as the minimal residual disease or multidrug resistance [60]. ESCs can represent the element from which the cancer is originated [61]. Microenvironment of chronic inflammation can be also responsible for malignant reprogramming of ESC [62]. This process seems to be more frequently occurring in bulge keratin K15/K19-positive cells than in interfollicular basal keratin K5/K14-positive cells and interfollicular suprabasal keratin K1/K10 and involucrin-positive cells [63]. The ESCs offspring located in the bulge can be activated and differentiated to the epidermal or hair follicle cellular lineage. When RAS/MAPK pathway is deregulated in mouse epidermis, squamous cell carcinoma can be expected in mouse model. In the case of PTCH/Shh signaling disturbance, development of basal cell carcinoma is likely in both mouse and human [62,63,64,65,66]. In context of cancer formation, increasing number of mitotic divisions of stem cells in different tissues reflects increased risk of cancer formation [67]. Tissue stem cells are present in body lifelong, however, the risk of their genetic damage becomes higher due to lower efficiency of DNA damage repair in elderly [68,69,70]. Thus, it can be assumed that the number of mitotic division of stem cells including the genetically damaged stem cells, is increasing in population scale. This is a direct consequence of rapid population aging in developed countries worldwide including Europe [71]. Due to these facts, an increasing part of the population in these countries will meet their cancer during their life can be hypothesized [72].

## 5. Tumor as Complex Organ with CSC Activity Stimulating Microenvironment

It was proposed by others that the complex structure observed in tumor has certain parallels to the structure of normal organs [73]. This also includes the assumption of existence of specific cancer microenvironment participating in CSC niche formation [74]. Almost thirty years ago, Harold Dvorak published a seminal article where he highlighted remarkable similarity between malignant tumors and wounds [75] (Figure 1). Really, tumor biology shares certain aspects with wound healing. In the case of epidermal carcinomas, the expanding tumor bud is similar to the pool of migrating and proliferating keratinocytes in course of reepithelization. Next to that, tumor stroma contains cell types and extracellular matrix similar to granulation tissue seen in healing wound. Tumor stroma also contains activated fibroblasts, inflammatory cells and sprouting blood capillaries [76]. All these cell types and their products stimulate migration and proliferation of epithelial cell which will result in either wound closure by reepithelization or in the aggressive growth and distant metastases of tumors. To highlight their specific features, fibroblasts in tumors are usually called cancer-associated fibroblasts (CAFs). CAFs frequently express smooth muscle actin. Based on their cytology, CAFs are mostly considered to be myofibroblasts [77]. CAFs produce extracellular matrix and also the proteinases for their degradation as well as numerous bioactive cytokines/chemokines/growth factors (Figure 1).

Similarly to granulation tissue, the tumor stroma is widely infiltrated by numerous immune cells. At least part of them exerts paradoxically strong protumorigenic properties [78]. In a broader sense, mutual interaction of skin microbiome and immune cells must also be considered even in activation or inhibition during epidermal carcinogenesis. This aspect can illustrate well the role of inflammation in cancer biology [79]. Similarly to some other tumors, prevention of skin inflammation by the nonsteroidal anti-inflammatory drug resulted in decreased risk of cutaneous squamous cell carcinoma [80].

Blood capillaries formed by endothelial cells are necessary for nutrients and gas exchange for tumor cells. Similarly to blood capillaries in granulations tissue, stromal capillaries lack complete wall and contain numerous fenestrations. This structural abnormality can result in accumulation of macromolecules in the tumor. This phenomenon could be employed in cancer therapy by drugs covalently attached to polymer carriers [81].

In conclusion, stroma reflects the biological properties of tumor. Multiple feedback mechanisms are engaged in regulation of local growth and distant metastases formation. This mechanism maintaining such dynamic equilibrium can be characterized as the coevolution of stroma with cancer cells [82].

## 6. CAFs as the Master Cell Type in Tumor Stroma

Classical CAFs are very similar to myofibroblasts of granulation tissue in healing wound as discussed in details above. The origin of CAFs is not well known yet. There is some evidence that CAFs are arising from local fibroblasts, mesenchymal stem cells, pericytes, endothelial cells and macrophages [83]. The most discussed possibility is the idea of epithelial to mesenchymal transition of cancer cells to CAFs. This was hypothesized and demonstrated in an animal experiment [84]. Moreover, HPV16 transformed lung epithelial cells are oncogenic and they acquire typical fibroblastic phenotype. When cocultured with normal human keratinocytes, normal keratinocytes acquire features very similar to cancerous cells [85]. On the other hand, human cancer cells grafted to nu/nu mice form tumors with stroma containing exclusively fibroblasts of the mouse origin, as was demonstrated by use of human-specific anti-vimentin antibodies [86]. Moreover, according to the mutational analysis in cancer of breast and ovary, the probability of formation of CAF from cancer cells is not expectable in clinically relevant extent [87]. Summarizing the data about the formation of CAF, their origin from local cells, mainly fibroblasts and mesenchymal cells is more probable than their formation from cancer cells. However, some effect of cancer cells after epithelial–mesenchymal transition on the tumor epithelium vicinity might be expected.

It is generally accepted that CAF stimulate cancer cell growth and metastatic behavior [76]. However, the opposite effect of these cells was also noted, for example in cancer of pancreas [88]. We demonstrated that fibroblasts isolated from basal cell carcinoma of the skin, squamous cell carcinoma of the head and neck and dermatofibroma of the skin stimulate expression of keratins 8 and 19 as well as of marker of mesenchymal cells—vimentin in cocultured keratinocytes [89,90,91]. This co-expression of keratins and vimentin and expression of transcription factor Snail in nuclei of remarkable proportion of keratinocytes under the influence of CAFs indicate that keratinocytes are in epithelial–mesenchymal transition, process so important for cancer cell metastases formation [92]. This stimulatory effect of CAFs on epithelial cells was observed in direct co-culture or in transwell system where both cell types were separated by microporous membrane. This setting suggests importance of paracrine effect of CAFs on epithelial cells. Of note, this activity of CAFs was well conserved during multiple passages *in vitro*.

Differences in transcriptional activity of CAFs were analyzed by robust quantitative approach such as microarray technology. This facilitated determination of more than 560 differently transcribed genes between normal fibroblasts and CAFs [93].

As mentioned above, local fibroblasts represent the main source for formation of CAFs. Cancer cells are able to activate normal dermal fibroblasts. This activation results in similar transcriptional profile to stroma of head and neck squamous cell carcinoma. Unfortunately, the same is true for normal human keratinocytes and their influence on dermal fibroblasts. In both cases, these stimulated fibroblasts significantly elevated expression of keratin 8 in normal human keratinocytes [56]. These *in vitro* activated fibroblasts are also remarkably similar to CAFs as demonstrated by proteomic approach [94]. Interestingly, this stimulation of normal dermal fibroblasts in response to presence of normal/malignant keratinocytes is only transient. This contrasts with long sustained activity of CAFs [95]. This difference between activity of CAFs and normal fibroblasts can be explained by epigenetic changes in CAFs such as DNA hypomethylation [96]. In opposite direction, effect of CAFs on normal/cancer epithelial cells seems to be very complex and hundreds of various gene products will participate in this intercellular crosstalk. Some of these mediators, such as BMP-4, IGF-2, IL-6, IL-8, CXCL-1, and TGF-β possess therapeutic potential and their inhibition can be applied in clinics in future [56,93,97,98,99].

Mesenchymal stem cells (MSCs) as a possible source of CAFs were hypothesized; MSCs stimulate breast tumor growth and metastases in mouse graft experiment [100]. Of note, bone/cartilage metaplasia in breast cancer stroma was really observed in human clinical samples [101]. It is necessary to highlight here that MSCs can differentiate to adipocytes, osteoblasts and chondroblasts. These observations suggest the tumor microenvironment as a compartment suitable also for MSCs. CAFs isolated from cutaneous basal cell carcinoma were able to influence embryonic mouse fibroblasts to express markers of pluripotency such as Nanog, Oct-4 and Sox-2. Moreover, these cells were consequently able to differentiate to adipocytes, osteoblasts and chondroblasts [102] confirming thus their acquired pluripotency equal to MSCs. The microarray analysis suggests IGF-2, FGF-7, leptin, nerve growth factor and TGF-β to be responsible for the change of mouse fibroblast to MSC-like elements. To conclude this paragraph, we have to emphasize that tumor microenvironment is active to epithelial cells but in comparable extent also to fibroblasts where it supports their properties similar to mesenchymal stem cells.

Extracellular matrix is produced and remodeled by CAFs and represents an important component of CSC niche [103]. Tenascin-C seems to be one of the most outstanding among plethora of other ECM components. It is accumulated in tumor stroma and also in wound. Tenascin-C stimulates aggressive growth of many tumor types [104]. Other important and far less known are endogenous lectins from the galectin family. Galectin-3 is significantly upregulated in organ fibrosis and galectin-1 in stroma of head and neck squamous cell carcinoma. Galectin-1 in HNSCS is accompanied by high presence of CAFs [105,106]. Galectin-1 is able to stimulate transformation of fibroblasts to myofibroblasts in higher extent than galectin-3 [52]. CAF and activated fibroblasts produce extracellular matrix and also impact proliferation of endothelial cells (HUVEC) [107]. These observations suggest participation of galectins in stromal reaction and so in CSC niche formation [108]. It is necessary to conclude here that molecules of extracellular matrix have a remarkable role in formation of niche for CSCs.

## 7. Malignant Melanoma: Microenvironment in Non-Keratinocytic Cutaneous Tumors

Keratinocytes represent the major, however not the only, cell type in human epidermis. The epidermis contains also minor cell populations such as infiltrating intraepidermal lymphocytes, antigen-presenting Langerhans cell, Merkel cells and melanocytes. The melanocytes are located in epidermal basal layer where they produce melanin important for protection of proliferating cells against UV-light damage. Melanocytes originate from neural crest. Neural crest derived stem cells are present in the bulge region of hair follicle during lifetime [20]. Malignant tumors arising from melanocytes are extremely aggressive. Therapeutic options in generalized disease are still rather limited. It is rather convenient in the context of this paper that their growth is highly regulated by the microenvironment. Melanoma cells injected into an embryo lose their malignant properties and migrate to the same target destinations as neural crest cells during normal embryonic development [109]. The clinical relevance of microenvironment in melanoma growth and progression was proposed [110]. The evidence commented above in paragraph “ESCs and their niche” clearly suggests existence of certain cooperation of ESCs and neural crest-originated stem cells within the bulge region of hair follicle. We demonstrated earlier that both melanoma cells and neural crest-derived stem cells significantly influence the differentiation status of normal human keratinocytes in similar manner. Keratinocytes under this influence are phenotypically similar to cancer cells or ESCs [111]. Further analysis demonstrated that FGF-2, CXCL-1, IL-8, and VEGF-A participate in the crosstalk of keratinocytes and melanoma or neural crest-originated stem cells, respectively. In opposite direction, keratinocytes support migration of melanocytes [112]. Fibroblasts prepared from malignant melanoma influence not only melanoma cells and keratinocytes but surprisingly even tumors of different origin, such as glioblastoma and breast cancer [113,114,115,116]. This highlights complexity of stromal interactions as well as certain conservation of these mechanisms in various tumor types. This conservation between various tumor types might be potentially important in future stroma targeted therapy. Tumor stroma targeting is still a relatively novel and not fully exploited concept in oncologic therapy. Tumor neoangiogenesis inhibition is probably the most classical paradigm in this type of therapy. Next to that, newly formed tumor vessels are also remarkably leaky due to their imperfect capillary walls. Based on this knowledge, multiple attempts of drug delivery directly to tumors were described with variable rates of success. Drugs as cytotoxic immunoconjugates or radioconjugates can easily extravasate from the leaky vessels selectively in tumor. Tumor stroma also hosts multiple types of immune cells. Immunotherapeutic approaches using e.g., tumor infiltrating lymphocytes or dendritic cells in cancer treatment have been evaluated during the last few decades. More recently, antibodies unblocking immune response are used in tumors escaping immune destruction (e.g., anti-CTLA-4 or anti-PD-1 in melanoma). Modification of local inflammatory environment in tumor stroma is also behind effect of drugs such as imiquimode or non-steroidal anti-inflammatory drugs. Specific targeting of CAFs is not recently in clinical use, however, early preclinical data are promising [53].
